# Biological Application of Novel Biodegradable Cellulose Composite as a Hemostatic Material

**DOI:** 10.1155/2022/4083477

**Published:** 2022-08-10

**Authors:** Xulong Zhu, Jianxiong Wang, Shuhan Wu, Tian Liu, Guangshuai Lin, Bin Shang, Jia Ma, Wudang Lu, Feifei Zhang, Jianhui Li, Jue Wang

**Affiliations:** ^1^The Key Laboratory of Biomedical Information Engineering of Ministry of Education, The Key Laboratory of Neuro-Informatics & Rehabilitation Engineering of Ministry of Civil Affairs; Institute of Health and Rehabilitation Science, School of Life Science and Technology, Xi'an Jiaotong University, Xi'an, Shaanxi 710049, China; ^2^Department of Surgical Oncology, Shaanxi Provincial People's Hospital, Xi'an, Shaanxi 710068, China; ^3^National Engineering Research Center of Health Care and Medical Devices, Xi'an, Shaanxi 710049, China; ^4^Xi'an Libang Pharmaceutical Technology Co., Ltd., Xi'an, Shaanxi 710000, China

## Abstract

Degradable hemostatic materials have unique advantages in reducing the amount of bleeding, shortening the surgical operation time, and improving patient prognosis. However, none of the current hemostatic materials are ideal and have disadvantages. Therefore, a novel biodegradable cellulose-based composite hemostatic material was prepared by crosslinking sodium carboxymethyl cellulose (CCNa) and hydroxyethyl cellulose (HEC), following an improved vacuum freeze-drying method. The resulting cellulose composite material was neutral in pH and spongy with a density of 0.042 g/cm^3^, a porosity of 77.68%, and an average pore size of 13.45 *μ*m. The composite's compressive and tensile strengths were 0.1 MPa and 15.2 MPa, respectively. Under in vitro conditions, the composites were degraded gradually through petite molecule stripping and dissolution, reaching 96.8% after 14 days and 100% degradation rate at 21 days. When implanted into rats, the degradation rate of the composite was slightly faster, reaching 99.7% in 14 days and 100% in 21 days. Histology showed a stable inflammatory response and no evidence of cell degeneration, necrosis, or abnormal hyperplasia in the tissues around the embedded material, indicating good biocompatibility. In the hemorrhagic liver model, the time to hemostasis and the total blood loss in the cellulose composite group was significantly lower than in the medical gauze group and the blank control group (*P* < 0.05). These data indicate that the novel cellulose composite is a promising implantable hemostatic material in clinical settings.

## 1. Introduction

Uncontrolled bleeding can be life-threatening, with hemorrhage being the second leading cause of death due to trauma [1–3]. Intraoperative wound bleeding is inevitable, and traditional hemostasis methods and improper wound protection during surgery may lead to vasoconstriction. Therefore, it is crucial to effectively manage the hemorrhage during such conditions to minimize patient morbidity/mortality. Local hemostasis is the most effective method for managing hemorrhage associated with trauma and surgery [4]. An ideal hemostatic material should be easy to handle, achieve rapid hemostasis, and be biocompatible and biodegradable, allowing it to be implanted over the wound within the site of surgery/injury to offer continued protection against hemorrhage. Degradable hemostatic materials have unique advantages in reducing the amount of bleeding, shortening the surgical operation time, and improving patient prognosis [5, 6]. At present, the primary biodegradable hemostatic materials used in the clinical application include chitosan, oxidized cellulose [7], collagen/gelatin sponge [8], and synthetic biomaterial [9–11]. However, with some associated problems, none of these materials are ideal. For example, collagen/gelatin sponges are of animal origin and are at risk of allergy and/or infection [12, 13]. The low pH value of oxidized regenerated cellulose may induce inflammation at the application site [14, 15]. Therefore, the development of a novel biodegradable hemostatic material carries clinical significance.

Polysaccharides are widely used and well-evaluated among the currently available hemostatic materials [6]. Cellulose is one such polysaccharide commonly used as a hemostatic material that mainly forms a gel-like substance to seal the wound and achieve physical hemostasis by rapidly combining with blood exuded from the wound surface. At the same time, it can also activate the coagulation pathway in the blood to accelerate wound hemostasis [16, 17]. Currently, the most commonly used commercially available cellulose hemostat is in the form of “oxidized regenerated cellulose (ORC),” which, according to a few studies, demonstrates the best clotting time [18, 19].

However, owing to its low pH, several studies have reported an intense foreign body response to ORC [20], associated with cytotoxicity [21], indicative of severe inflammatory reaction that can affect the healing process [15, 20]. Krízová et al. suggest that ORC could reduce the pH value around the damaged tissue; such a high acid environment could enhance the inflammatory response in the injured tissue, which finally delays the wound healing [22]. Herein, our research group fabricated a novel, implantable cellulose composite hemostatic material for an intraoperative or prehospital emergency by mixing and modifying two different types of cellulose and optimizing the preparation process. This study is aimed at evaluating its physical properties, biodegradability, and hemostatic effect under *in vitro* and *in vivo* conditions, providing the preliminary experimental foundation for its clinical translation.

## 2. Materials and Methods

### 2.1. Ethics Statement

The study protocol and the use of animals in the research were approved by the ethics committee of Xi'an Jiaotong University and carried out following the guidelines of the laboratory animal care committee, Medical Department, Xi'an Jiaotong University.

### 2.2. Materials and Reagents

Sodium carboxymethyl cellulose (CMC-Na) and hydroxyethyl cellulose (HEC) were purchased from the Shandong Yousuo Chemical Technology Co., Ltd. Forty-eight Kunming female Sprague-Dawley (SD) rats, weighing 195–252 g, were purchased from the Experiment Animal Center, Medical School of Xi'an Jiaotong University (Xi'an, China). All animals were acclimatized for one week before the experiment in the Central Laboratory of Shaanxi Provincial People's Hospital [license number SYXK (Shaanxi) 2016-006].

### 2.3. Preparation and Process Improvement of Novel Hemostatic Material

The degradable cellulose composite material was fabricated by an improved vacuum freeze-drying method involving batching, dissolution, cross-linking, ultralow temperature freezing, and sublimation drying. Briefly, sodium carboxymethyl cellulose (10 g/ml) and hydroxyethyl cellulose of the same concentration was dissolved in 100 ml distilled water in a 500 ml flask; the mixture was left on a magnetic stirrer (400 r/min) at 60°C for 1 h. After the crosslinking process, the mixture was cooled down to room temperature, and pH was measured and put into a circular mold (petridish of 8.5 cms in diameter) for standby application. Then, the mold was transferred to the ultralow temperature freezer (-50°C) for 5 h. Finally, the frozen cellulose composite was freeze-dried to a film using a vacuum freeze dryer at -15°C for 10 h and 25°C for 8 h. The resulting cellulose composite material, 8.5 cms in diameter and 0.4 cms in thickness, was wholly dried in a vacuum until a constant weight was observed in three consecutive measurements, sterilized under ultraviolet radiation, and stored in a vacuum at 4°C until further use.

### 2.4. Characterization of the Cellulose Composite Hemostatic Material

Gross observation of the cellulose composite material was done, followed by analyzing its physical properties (density and porosity) and mechanical properties (compressive strength and tensile strength). Further, the surface structure of the prepared composite material, along with its pore size, was studied using scanning electron microscopy (TESCAN).

### 2.5. Degradation *In Vitro*

The cellulose composite material was cut into 3 cm × 3 cm sizes under aseptic conditions and weighed separately, then immersed in 20 ml of phosphate-buffered solution (pH = 7.4). Degradation was carried out on a shaking incubator at a temperature of 37°C, and fresh phosphate buffer (Merck) was changed regularly. The samples were removed from the incubator and measured. The weight of each sample was at predetermined time points of 1, 3, 7, 11, 14, and 21 days, after which the overall integrity of the material was observed. The soaking solutions were measured for their pH, centrifuged at 3000 rpm for 10 min, and the supernatant was filtered with a 0.22 *μ*m filter membrane to collect solid residues, which were vacuum dried until a constant weight was observed on an electronic balance. The degradation rate was then calculated using the formula: degradation rate = (initial weight − residual weight)/initial weight × 100. The average of four weight measurements was recorded.

### 2.6. Tissue Compatibility and Degradation *In Vivo*

Sixty SD rats were randomly divided into 6 equal groups according to time points of sacrifice (1, 3, 7, 11, 14, and 21 days) to study the degradation rate *in vivo*. Of the 10 rats per group, 5 rats were randomly allocated to the implant group, while the other 5 rats served as the sham control. The entire procedure was conducted in a sterile biological cabinet. The rats were anesthetized by intraperitoneal injection of pentobarbital sodium. The cellulose composite was cut into two pieces (1 × 1 cm each) and implanted into the subcutaneous fascia tissue on both sides of the rat's spine. The rats were sacrificed, and the composite material and surrounding subcutaneous tissue, or the tissue around the surgical site for the control group, were harvested at 1, 3, 7, 11, 14, and 21 days after implantation, embedded in paraffin, sectioned, and hematoxylin-eosin (HE) stained for histological analysis.

### 2.7. Hemostasis Experiment *In Vivo*

Fifteen SD rats were randomly divided into 3 groups, with 5 in each group. The rats were anesthetized with an intraperitoneal injection of pentobarbital sodium and were fixed in the supine position on a small operating table before placing a midline cut to expose the liver. A bleeding wound of approximately 20 mm in length and 2 mm in depth was created by excising a part of the left lobe of the liver. The rats were then allowed to bleed freely for 3 seconds, after which one group of rats was treated with cellulose composite material (100 mg), while the rats in the second group received the same quantity of medical gauze to stop bleeding and observe the indexes. The rats in the third group received no treatment for their bleeding wounds and served as the blank control.

### 2.8. Hemostasis Time

Hemostatic material was placed on the bleeding wound, over which a layer of plastic film was placed to avoid adhesion. The wound was observed every 30 seconds until the bleeding completely stopped. The final time of complete hemostasis was recorded for future analysis.

### 2.9. The Amount of Blood Loss

The electronic balance was used to accurately weigh the hemostatic materials (*m*_0_) and dry cotton balls (MD) to wipe off the spilled blood in advance. After achieving hemostasis, the hemostatic materials (*m*_1_) and cotton balls (*m*_*w*_) were reweighed in a sealed container. The amount of blood loss was then calculated by the formula mBL (*g*) = (*m*_1_ + *m*_*w*_) − (*m*_0_ + *m*_*d*_) or mBL = *m*_*w*_ − *m*_*d*_. The average of three independent measurements was considered the final amount of blood loss, and the average of six rats was taken as the final test result, unit (g/Kg).

### 2.10. Statistical Analysis

Data were analyzed using SPSS 20.0 (IBM) package. All data were presented as mean ± standard deviation (SD). Between-group differences during each experiment were analyzed using analysis of variance (ANOVA). Statistical differences between samples were performed with a significance level of *P* < 0.05.

## 3. Results

### 3.1. Characterization of the Novel Cellulose Composite Hemostatic Material

**Figure 1:**
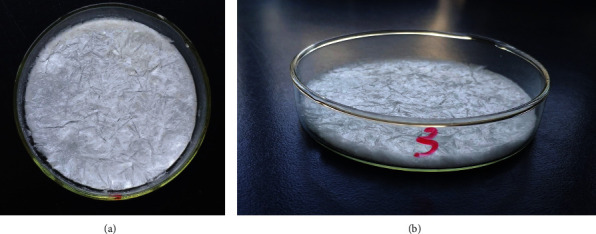
The gross morphology of cellulose composite, a degradable hemostatic material.

The pH of the cellulose composite mixture prior to freeze-drying was 7-8. The cellulose hemostatic composite sponge prepared in circular disk-shaped molds was white, spongy, and pliable with good flexibility and slightly rough on the upper and lower surfaces (Figures 1(a) and 1(b)).

Further, the material exhibited a porosity of 77.68%, density of 0.042 g/cm^3^, compressive strength of 0.1 MPa, and tensile strength of 15.2 MPa.

**Figure 2:**
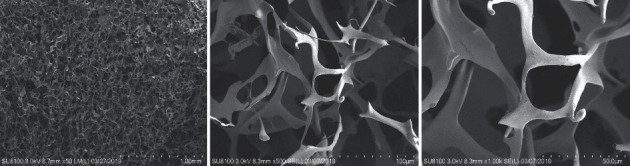
Scanning electron microscope observation of cellulose composite hemostatic material.

Images from scanning electron microscopic (SEM) showed that the surface morphology of the cellulose composite sponge was dense with no phase separation, indicating the success of the optimized process. The surface of the membrane material showed irregular porous network structures, with an average pore size of 13.45 *μ*m and interlaced fibrous features which help enhance the tensile strength (Figure 2).

### 3.2. Degradation *In Vitro*

#### 3.2.1. Gross Observation of the Material

The newly synthesized composite material was white, opaque, and flexible. From day 1 onward, the material significantly swelled in the phosphate-buffered solution. From day 1 to day 3, the material continued to swell, and the erosion that could be seen with the naked eye gradually increased with no noticeable change in color. The material gradually shifted from solid-state to semisolid and semigel state. After 1 week, the material became sticky, gelatinous, and soft, which showed focal detachment and irregular stripes and holes on the surface. On the 11th day, the material was swollen completely, and most of it was peeling off and dissolved, yielding thin and crispy material after vacuum drying; after 14 days, the material lost its primitive form completely, and some acceptable granular debris could be seen in the phosphate-buffered solution. By the 21^st^ day, the materials were invisible to naked eyes with no solid residues, illustrating complete uniform dissolution in the simulated body fluid.

#### 3.2.2. Degradation Rate *In Vitro*

**Figure 3:**
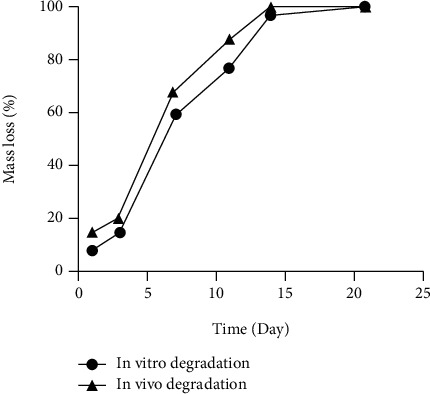
Degradation rate of cellulose composite hemostatic materials in rats and phosphate-buffered solutions.

The degradation of cellulose composite material presented a certain regularity (Figure 3). The average degradation rate of the experimental samples was 8.2% on day 1, 15% on day 3, 58.5% on day 7, 77% on day 11, 96.8% on day 14, and 100% by day 21. The pH of the soaking solutions was 7-8 at all the measured time points.

### 3.3. Degradation *In Vivo* and Tissue Biocompatibility

#### 3.3.1. Gross Observation

**Figure 4:**
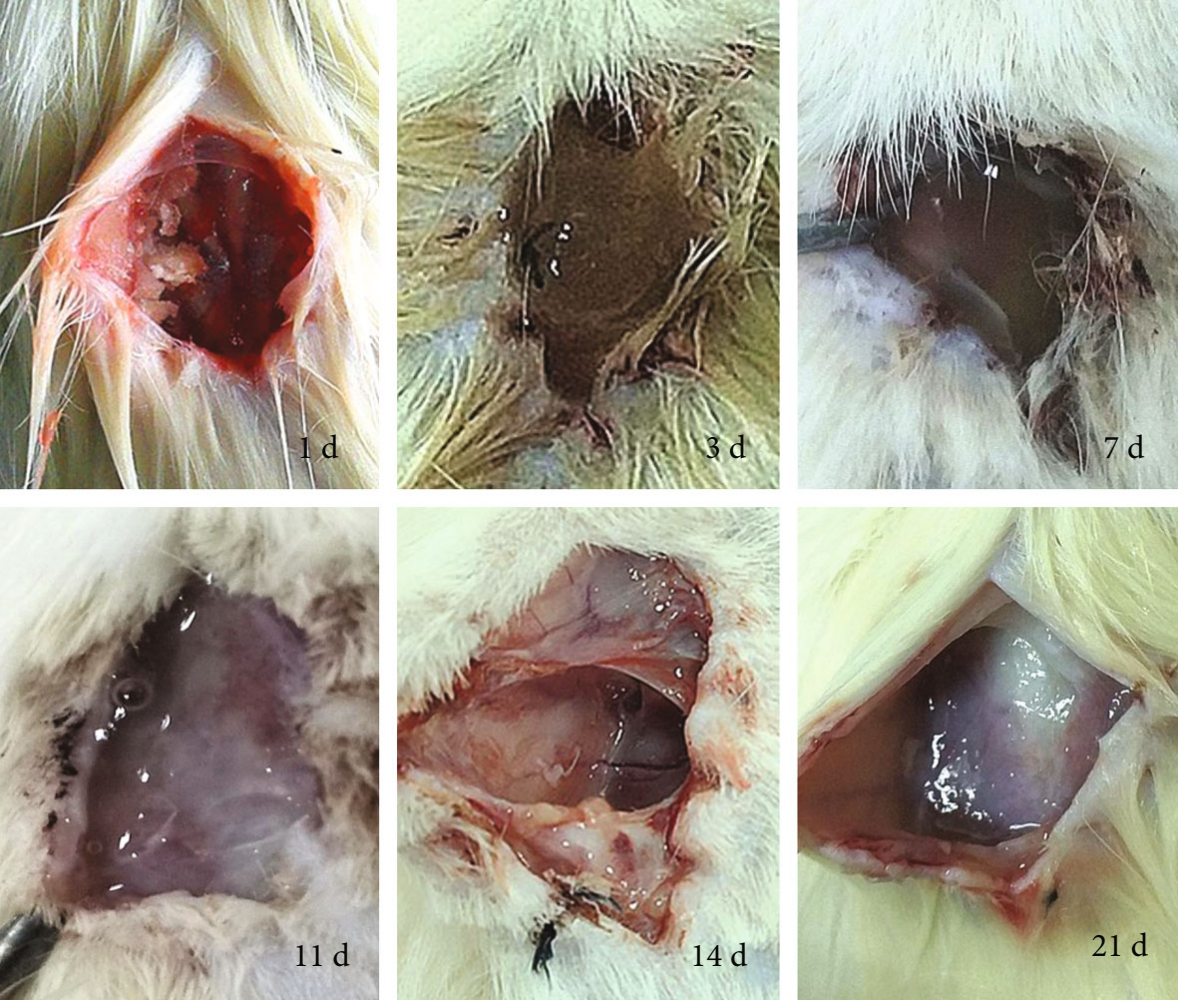
Morphological changes in the degradation of cellulose composite in rats. After degradation.

All the rats started drinking and eating after the surgery and exhibited regular activity until the end of the experiment with no postsurgery mortality. There was no redness, ulceration, or secretion observed around the surgical site in both implant and control group rats, and the hairs at the surgical site grew normally without induration. On the first postoperative day, the wounds healed well, and the material was found to be in semisolid and semigel state, pale yellow without redness or abnormal secretions in the surrounding tissues. Three days after the operation, the incisions were almost healed, and the hair around the incision began to grow. It was observed that the implantation sites were gelatinous, viscous, and in close approximation with adjacent tissues. On the 7th day, the back incisions were healed completely, classifying them as first-grade healing, and the tissues surrounding the material began to integrate well with the thin, pale yellowish, and gelatinous material. From day 11 to 21, the materials gradually decreased in size and with no signs of inflammation around the tissues. By 21 days of implantation, there seemed to be no residue visible to the naked eye. The changes in the materials are shown in Figure 4.

For 1 day *in vivo*, the solid membrane of cellulose composite material began to appear in semisolid and semigel state, and no abnormal secretion was observed. Three days after surgery, all the implanted materials were observed to be gelatinous and viscous. 7 days after the operation, the materials were thin, yellowish, and gelatinous, and the tissue was not easily separated from the material. 11 days after implantation, the gelatinous membrane in the group was significantly thinner and reduced in size than before, and no abscess or necrotic tissue was observed. Most surrounding tissues and materials in the 14-day group were fused, with apparent degradation and absorption. The materials were further absorbed in the 21-day group, and no residue was visible to the naked eye.

#### 3.3.2. Degradation Rate *In Vivo*

The degradation process of the cellulose composite material samples in rats was similar to that of *in vitro* but slightly faster (Figure 3). The average degradation rate of the cellulose composite *in vivo* was 15.2%, 20.6%, 68%, 88.0%, and 99.7%, respectively, on days 1, 3, 7, and 14. The material was completely dissolved on the 21st day, making it impossible to measure the sample weight, and thus, the average degradation rate was considered 100%.

#### 3.3.3. Histological Evaluation

**Figure 5:**
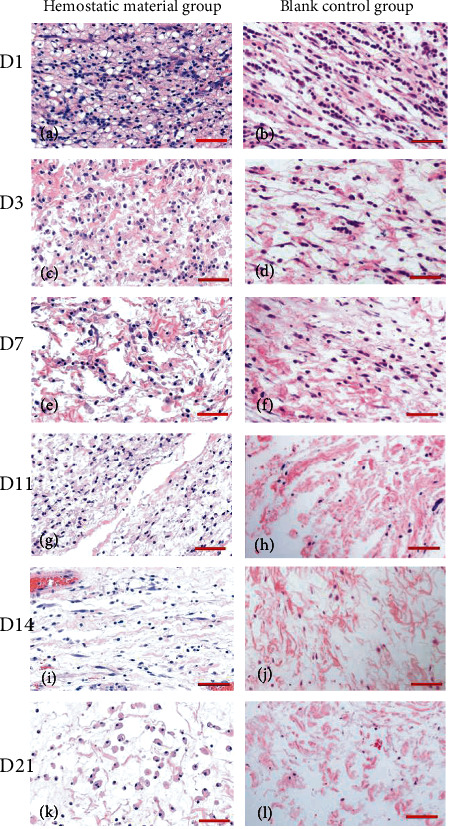
Histological evaluation of implantation in rats.

Histopathological findings showed some inflammatory cellular infiltration in both implant and control groups. At 1 day (Figure 5(a)) and 3 days (Figure 5(c)) postimplantation, the pathological sections of the material and surrounding tissues showed infiltration of inflammatory cells that mainly comprised neutrophils, lymphocytes, and fewer foamy macrophages, with an unclear boundary from the surrounding connective tissues. The histology of the control group also showed infiltration of inflammatory cells that mainly comprised of lymphocytes and a few neutrophils (Figures 5(b) and 5(d)). After 7 days (Figure 5(e)) and 11 days (Figure 5(g)), the number of inflammatory cells decreased, although several foamy macrophages and lymphocytes were still visible in the implant group. A fibrous connective tissue capsule was seen around the subcutaneous implant. In the control group, fibrous tissue hyperplasia was found in subcutaneous tissue with fewer lymphocytes and macrophages infiltrated, while neutrophils were not visible anymore (Figures 5(f) and 5(h)). The intensity of inflammation gradually decreased in both groups. At 14 days (Figure 5(i)) and 21 days (Figure 5(k)), the macrophages persisted in the implant group, which played a positive role in the wound healing process. The demarcation between the materials and surrounding tissues was fuzzy, and the fiber layer was thinning after 21 days. The control group also exhibited fewer macrophages at these time points (Figures 5(j) and 5(l)). None of these samples showed cell degeneration, necrosis, or abnormal hyperplasia.

### 3.4. Hemostasis Ability *In Vivo*

#### 3.4.1. Hemostasis Time

In the hemostasis experiment of the liver bleeding wound model, the cellulose composite material rapidly adhered and tightly covered the wound surface, after which its color changed from white to dark red, while the material changed from sponge to gel-like consistency. During the whole process of hemostasis, the cellulose composite material tightly adhered to the wound surface and was hard to peel off. However, the medical gauze could only contact the bleeding surface with the support of physical compression, and when it absorbed the blood to a certain extent, it lost the hemostatic effect because of the failure to adhere to the wound. The wound stopped bleeding in the blank control group, via a natural blood-clotting mechanism.  Table 1 shows the hemostasis time and the amount of blood lost in all three groups.

The time to complete hemostasis in the cellulose composite material group was 3.11 min, which was markedly lower (*P* < 0.05) compared to the medical gauze (4.58 min) and the blank control group (5.77 min). The time to complete hemostasis for the sterile gauze group was also markedly lower than the blank control group (*P* < 0.05).

#### 3.4.2. Bleeding Volume

The total blood loss after applying the cellulose composite material was 12.98 g/kg, which was significantly lower (*P* < 0.05) than that observed in the sterile gauze (17.89 g/kg) and blank control (22.66 g/kg) group. A statistically significant difference was also observed between the medical gauze group and the blank control group (*P* < 0.05) for the mean blood loss.

## 4. Discussion

Any surgery, especially open surgeries, including tumor excision, often causes bleeding of the organs or tissues during and after the procedures. Electrocoagulation and suture ligation can be convenient for intraoperative hemostasis in routine clinical applications. However, there is an increasing trend toward using degradable hemostatic materials for local packing and compression hemostasis [23]. Several hemostatic agents, such as cyanoacrylate-based glues, gelatin sponges, fibrin compounds, polysaccharides, are available on the market. However, toxic substances released from cyanoacrylic hemostatic materials during the degradation process *in vivo* may cause inflammatory reactions and even delayed wound healing [24]. Gelatin and fibrin compounds are derived from animal tissues, posing the risk of allergic reactions [25]. For these reasons, the polysaccharide hemostatic material, a natural bioabsorbable polymer that has many desirable properties such as good biodegradability, good biocompatibility, and low cost, along with being abundantly available, is increasingly becoming a research hotspot of degradable hemostatic materials [26–31]. The most studied polysaccharide hemostatic materials include cellulose, chitosan, and starch [32, 33]. Among them, cellulose could form a soft gel-like “pseudoclot” around the injury that acts as a barrier to occlude the blood flow while also can activate the intrinsic coagulation pathway to accelerate the blood coagulation process [34, 35].

With this background, in this study, we developed a novel cellulose composite material by cross-linking carboxymethyl cellulose and hydroxyethyl cellulose polymers and subjecting it to an improved vacuum freeze-drying method. The material demonstrated good flexibility and high tensile strength, which could be convenient for intraoperative hemostasis in different clinical situations. In our experiment, the cellulose composite material exhibited good water absorption and swelling behavior and wholly degraded in simulated body fluids. The weight loss of materials in the initial stage was the greatest, where the surface erosion phenomenon was visible. Observing the degrading composite under SEM showed that the material changed from an irregular fibrous structure before degradation to a curly contractive one, and then, the cracks and crevices also appeared on the sample surface. Gross morphology showed that the state of material changed from solid to semisolid and semigel state, gradually got thinner, and then, eventually disappeared, indicating that the composite degraded through small molecules stripping dissolution.

Further, the composite material was wholly absorbed in experimental animals without inducing severe adverse reactions. Along the duration of the degradation time *in vivo*, the inflammatory response tends to be stable and alleviated due to the absorption of materials, indicating good biocompatibility. In this study, the degradation time *in vivo* of cellulose polymer materials was about 14 days, which shows an improved time compared to the ones reported in the literature [32]. This degradation time is moderate and poses no risk of foreign body residue and granuloma formation that would otherwise be seen in materials with a long degradation time [36, 37]. There was no inflammatory reaction such as redness, swelling, and pain around the implanted material. While histologic studies of materials have shown a nonspecific aseptic inflammatory response, an early acute aseptic inflammatory response could have been caused by surgical stimulation and fine degraded particles of composite material.

From the biomaterial perspective, the implantation of hemostatic materials in experimental animals evoked a systemic reaction of trauma, including inflammation, wound healing, and foreign body reaction. This kind of inflammation could be attributed to a nonspecific inflammatory response. The early inflammatory reaction in the sham operation group was mainly caused by traumatic stimulation such as operation and mechanical injury, which gradually disappeared with a resolution of inflammation and tissue repair. However, the inflammatory response in the cellulose composite group was more prevalent compared to the control group, with the most likely reason being that the degradation products of monomers or polymers may cause the aggregation of inflammatory cells. Over the time of degradation and the absorption and metabolism of hemostatic material, the inflammatory reaction in the experimental group gradually regressed, which was not significantly different compared to the control group.

We found the cellulose composite material exhibited superior hemostasis performance in rat liver hemorrhagic wound model compared to medical gauze. The difference in blood loss and the time to hemostasis between the two groups was statistically significant. In this study, the novel cellulose composite material shows a good hemostasis effect, biocompatibility, and degradability. In addition, it also demonstrated good physical and mechanical properties, which can be further tuned by altering the ratio of the cellulosic polymers or material structure to meet clinical needs better. Also, being implantable biomaterial, the cellulose composite can be optimized to carry drugs, like hemostatic agents and antitumor drugs, to play dual roles in achieving both hemostasis and tumor dissemination, towards which our future research is warranted. Moreover, the comprehensive source of its availability makes cellulose composite one of the harmless, environmentally friendly, and cost-effective materials to be developed on an industrial scale to meet clinical needs.

## Figures and Tables

**Table 1:** Hemostasis time and amount of blood loss in rat liver bleeding wound model treated with different hemostatic materials. ^∗^*P* < 0.05.

Group	The bleeding time (min)	The bleeding (g/kg)
Cellulose composite	3.11 ± 0.70^∗^	12.98 ± 2.28^∗^
Medical gauze	4.58 ± 0.42	17.89 ± 4.32
Blank control group	5.77 ± 1.00	22.66 ± 0.93

## Data Availability

The labeled dataset used to support the findings of this study are available from the corresponding author upon request.
